# A red seaweed *Kappaphycus alvarezii*-based biostimulant (AgroGain^®^) improves the growth of *Zea mays* and impacts agricultural sustainability by beneficially priming rhizosphere soil microbial community

**DOI:** 10.3389/fmicb.2024.1330237

**Published:** 2024-04-02

**Authors:** Nagarajan Nivetha, Pushp Sheel Shukla, Sri Sailaja Nori, Sawan Kumar, Shrikumar Suryanarayan

**Affiliations:** Research and Development Division, Sea6 Energy Private Limited, Centre for Cellular and Molecular Platforms, NCBS-TIFR Campus, Bengaluru, India

**Keywords:** sustainable agriculture, red seaweed-based biostimulants, *Kappaphycus alvarezii*, metagenome, rhizosphere microbiome, soil health, *Zea mays*

## Abstract

The overuse of chemical-based agricultural inputs has led to the degradation of soil with associated adverse effects on soil attributes and microbial population. This scenario leads to poor soil health and is reportedly on the rise globally. Additionally, chemical fertilizers pose serious risks to the ecosystem and human health. In this study, foliar sprays of biostimulant (AgroGain/LBS6) prepared from the cultivated, tropical red seaweed *Kappaphycus alvarezii* increased the phenotypic growth of *Zea mays* in terms of greater leaf area, total plant height, and shoot fresh and dry weights. In addition, LBS6 improved the accumulation of chlorophyll a and b, total carotenoids, total soluble sugars, amino acids, flavonoids, and phenolics in the treated plants. LBS6 applications also improved the total bacterial and fungal count in rhizospheric soil. The V3-V4 region of 16S rRNA gene from the soil metagenome was analyzed to study the abundance of bacterial communities which were increased in the rhizosphere of LBS6-treated plants. Treatments were found to enrich beneficial soil bacteria, i.e., Proteobacteria, especially the classes Alphaproteobacteria, Cyanobacteria, Firmicutes, Actinobacteriota, Verrucomicrobiota, Chloroflexi, and Acidobacteriota and several other phyla related to plant growth promotion. A metagenomic study of those soil samples from LBS6-sprayed plants was correlated with functional potential of soil microbiota. Enrichment of metabolisms such as nitrogen, sulfur, phosphorous, plant defense, amino acid, co-factors, and vitamins was observed in soils grown with LBS6-sprayed plants. These results were further confirmed by a significant increase in the activity of soil enzymes such as urease, acid phosphatase, FDAse, dehydrogenase, catalase, and biological index of fertility in the rhizosphere of LBS6-treated corn plant. These findings conclude that the foliar application of LBS6 on *Z. mays* improves and recruits beneficial microbes and alters soil ecology in a sustainable manner.

## 1 Introduction

Soils are an important part of the natural environment as they functionally integrate atmosphere, lithosphere, hydrosphere, and biosphere to achieve climate sustainability, healthy food production, and an adaptable natural environment (Larkin, 2015; Lehmann et al., 2020; Panagos et al., 2022). The well-being of soils is directly proportional to agricultural sustainability and environmental quality (Larkin, 2015). In general, soil health is defined as the ability of the soil within various ecosystems to improve biological productivity and enhance environmental quality and support the health of plants and animals (Kibblewhite et al., 2008; Lehmann et al., 2020). The physicochemical properties, micronutrient/macronutrient content, enzymes, and microbial diversity of soils are factors which are also increasingly being recognized as essential to sustainable agriculture productivity (Liao et al., 2018). Rapid responses and sensitivity to any environmental changes make microbes early warning indicators of soil health, i.e., the canary in the coalmine scenario (Wu et al., 2020). Bacteria and fungi are the main constituents of the soil microbial community and play a vital role in the decomposition of crop remains and maintenance of organic matter (Fabian et al., 2017). They also help in mineralization and nitrogen, carbon, and sulfur cycling (Mhete et al., 2020). Soil enzymes, which are the direct manifestation of a healthy soil microbial population and are extracellular and endogenous, are thought to be an indication of changes in soil quality (Bastida et al., 2008). These enzymes play crucial roles in controlling ecosystem processes for long-term soil nutrient cycling and breakdown and transformation of organic and inorganic nutrients into biologically available forms (Dotaniya et al., 2018). The metabolic functions of soil microorganisms depend on the contribution of soil enzymes, biochemical changes, material conversion, and redox and energy metabolisms (García-Ruiz et al., 2008).

To meet the nutritional requirements of a continuously increasing global population, agricultural practices generally depend on the long-standing, overuse of chemical fertilizers, which have adversely impacted the structure of soils and their microbial communities. As a result, there is a general deterioration of the soil due to dysfunctional microbial diversity and subpar microbial activity (Chaparro et al., 2012). This serious situation, which has hitherto unrecognized and ignored, necessitates the development of more sustainable, environmentally friendly strategies for increasing agricultural productivity. Natural agricultural inputs known as plant biostimulants have been shown to increase many crops’ abilities to withstand abiotic stress and absorb nutrients more effectively (Calvo et al., 2014; Van Oosten et al., 2017; Bulgari et al., 2019; Shukla et al., 2019; El Boukhari et al., 2020; Shukla and Prithiviraj, 2021; Shukla et al., 2023). A major class of the current regulatory category of plant biostimulants is derived from a variety of seaweeds, predominantly members of the Ochrophyta (i.e., brown seaweeds) (Shukla et al., 2016). In general, those seaweeds selected as raw materials for the manufacturing of biostimulant extracts are rich sources of a very wide range of natural and hydrolysis-induced bioactive compounds, as the seaweeds used are generally grown in extreme marine habitats (Potin et al., 1999; Shukla et al., 2016; Pereira et al., 2020; Shukla et al., 2021). The biostimulants derived from seaweeds are known to increase plant growth by inducing stress tolerance and efficient nutrient utilization (Shukla et al., 2019; Deolu-Ajayi et al., 2022; Trivedi et al., 2023).

The bioactive component present in seaweed-based biostimulants is also known to improve soil health by modulating the rhizosphere microbial community of crops, such as tomato, pepper, corn, and rice (Renaut et al., 2019; Shukla et al., 2019; Zhou et al., 2019; Hussain et al., 2021; Chen et al., 2022). Shifts in soil microbial community structure, improved microbial count, and available nutrients were observed as a result of applications with seaweed extracts such as the brown seaweeds *Ascophyllum nodosum*, *Durvillaea potatorum*, and *Sargassum horneri* and the red seaweed *Kappaphycus alvarezii* (Wang et al., 2018; Hussain et al., 2021; Trivedi et al., 2022). The rhizospheric region is extremely rich in microbial diversity as plants alter the constituents of their root exudates (Olanrewaju et al., 2019; Vives-Peris et al., 2020), and some biostimulants were reported to alter the production of primary and secondary metabolites in plants (Shukla et al., 2019).

The red alga, *Kappaphycus alvarezii*, is a cultivated eucheumatoid, which is mainly grown in tropical marine regions. Its biomass has a number of commercial applications by way of the extraction of kappa carrageenan, which is broadly used in the highly processed foods, beverages, nutraceuticals, para-pharmaceutical, and aquaculture industries. Seaweed and its extracts have recently used in the agricultural sector (Shukla et al., 2023). Various bioactive compounds were detected in the aqueous sap of *K. alvarezii* (Vaghela et al., 2022), and it is known to increase stress tolerance and yield in different crops (Layek et al., 2018; Patel et al., 2018; Trivedi et al., 2018; Kumar et al., 2020). AgroGain^®^ (product code: LBS6) is a commercial biostimulant with differentiated active ingredients, which is extracted from *K. alvarezii*, and is prepared by combining different bioactive fractions present in juice-extracted pulp and acid hydrolysate from pulp after juice extraction, following proprietary method developed at Sea6 Energy Private Limited, Bengaluru, India (Nori et al., 2019, US10358391B2; Shukla et al., 2023). The bioactive components of AgroGain^®^ include sulfated galacto-oligosaccharides with a specific molecular weight range of 400–10,000Da (Nori et al., 2019; Shukla et al., 2023) and is known to increase growth of plants and reduce harmful effects of different abiotic stresses on plants (Ravi et al., 2018; Arun et al., 2019; Banakar et al., 2022; Shukla et al., 2023). The foliar application of AgroGain^®^ is reported to induce plant growth by modulating the expression of genes involved in developmental and physiological pathways (Shukla et al., 2023). A foliar spray of biostimulant derived from *K. al*var*ezii* in rice decreases the inhibiting effect of fungicides by adjusting antioxidative pathways in different ways (Banakar et al., 2022). Similarly, AgroGain^®^ enhanced the nutrient assimilation, growth, and yield of banana and cucumber (Ravi et al., 2018; Shukla et al., 2023). However, hitherto, there is very limited knowledge about how foliar spray applications of these extracts induce changes in the soil microbial communities. In this study, an attempt was made to understand how foliar spray applications of AgroGain^®^ influenced the soil health and associated microbial community and their concomitant relationship with crop yield. In addition, morphological, physiological, and photosynthetic parameters were evaluated to study the various effects of AgroGain^®^ (LBS6) foliar spray on the growth of *Zea mays* plants.

## 2 Materials and methods

### 2.1 Source of seaweed extract and seeds

In the present investigation, AgroGain^®^ (LBS6), a novel class of *Kappaphycus alvarezii*-based biostimulant, was used. Chemical composition of LBS6 was previously published by Shukla et al. (2023). Seeds of sweet corn (*Zea mays*, variety of Hybrid Mithas) were procured from Nongwoo Seeds India Pvt. Ltd., Bengaluru, India.

### 2.2 Study design and soil sample collection

Experimental details are shown in Supplementary Figure S1. In brief, a greenhouse potting trial was conducted to study the effect of LBS6 on soil health and microbial activity. In total, 6 inch pots were filled with 2 kg of soil. The soil used in this experiment was collected from a fallow arable land, without cultivation for 2 years. It was red, sandy loam soil with a pH of 7.37 and EC of 186.1 μS/cm. The soil was sieved with a 2 mm sieve, mixed thoroughly, and then used for the experiment. Two seeds were planted in each pot at a consistent depth of 2 cm. Overall, 6 days after sowing (DAS), the seedlings were thinned to maintain one seedling per pot, and uniform seedlings were selected for the experiment. The seedlings were drenched with 50 mL of ½ Hoagland solution (Himedia, India) as the nutrient source at 7 and 21 DAS. Plants were sprayed with 20 mL of 1 mL. L^−1^ of LBS6 containing 0.01% Tween 20 after 14 DAS and with 40 mL of 1 mL. L^−1^ of LBS6 after 28 DAS for a second spray. The plants sprayed with 0.01% Tween 20, served as a control. The soil in the pots was covered with aluminum foil during foliar spray to prevent spraying of LBS6 to the soil. The plants were irrigated with 100 mL of water every 2 days. The morphological, physiological, and biochemical parameters were recorded on plants harvested at 35 DAS. The plants were arranged in a randomized block design, and the experiment was carried out on a metal bench in a greenhouse maintained at 24 ± 3°C by circulating the air using the fan and evaporative pad cooling system linked with a temperature sensor. The plants were grown under a natural day-light cycle. Each experiment comprised 10 replicates which were repeated thrice.

Before sowing of seeds, soil samples collected from un-planted pots were termed as bulk soil (BS). For treatments, the plants were gently dislodged from the pots, and the soil adhered to the roots was sampled for microbial diversity and soil health analysis. The samples were collected at 31 and 35 DAS from the rhizospheric zone of the plants sprayed with water and 0.01% Tween 20 (control) and 1 mL. L^−1^ of LBS6 with 0.01% Tween 20 as follows: soil control 1 (SC1, soil collected 3 days after second spray from the roots of control plants), soil control 2 (SC2, soil collected 7 days after second spray from the roots of control plants), soil treatment 1 (ST1, soil collected 3 days after second spray from the root of LBS6 sprayed plants), and soil treatment 2 (ST2, soil collected 7 days after second spray from the root of LBS6 sprayed plants). At each time point, samples were collected from the upper 15 cm of soil of 10 pots, the soil adhering to the roots of plants from the rhizosphere region. The soil samples were pooled, sieved, homogenized, and stored at 4°C for further analysis. The soil samples were collected from the rhizospheric region of 10 plants in each experiment, and every experiment was independently repeated three times.

### 2.3 Phenoptypic observations

The height of the plants and number of leaves were recorded at 35 DAS from 10 individual plants for each treatment. Increase in leaf area was recorded using WinFolia Basic. The shoots and roots were separated from the hypocotyl region, and shoot fresh weight was recorded using a fine weighing scale (Sartorius, Germany). The dry weight of shoots was recorded after drying in an oven at 70°C for 72 h. These morphological parameters were collected from three independent experiments. Each experiment consists of 10 replicates (*n* = 30).

#### 2.3.1 Estimation of pigments

Chlorophyll a and b and carotenoid contents from leaves of the control and LBS6-treated plants were measured according to the protocols described by Lichtenthaler (1987). The sixth leaves from the bottom of the 35-day-old plants were selected for pigment analysis. A leaf sample weighing 100 mg was crushed with a mortar and pestle in 500 μL of chilled methanol. The resulting solution was then centrifuged (10,000 rpm, 10 min at 4°C), and the extraction process was repeated twice. The extracts were pooled, and the volume was adjusted to 1.5 mL. The absorbance was recorded at three different wavelengths (470, 652.4, and 665.2 nm). The following equations were used for calculating chlorophyll and carotenoid content:

$Chl\;a=16.72\;A_{665.2:}9.16\;A_{652.4}$

$Chl\;b=34.09\;A_{652.4}:15.28\;A_{665.2}$

Carotenoids=1,000A470−1.63Chla−104.96Chlb/221

#### 2.3.2 Measurement of chlorophyll fluorescence and photosynthesis-related parameters

Photosynthesis-related parameters were recorded in leaves of the LBS6 and control plants by MultispeQ V2.0 (PhotosynQ LLC, East Lansing, MI) and PhotosynQ platform using Photosynthesis Rides 2.0.^1^ Chlorophyll fluorescence (ChlF) was assessed on the fully expanded leaves of 35-day-old corn plants at the same physiological position (second and third). The following parameters were observed in this study: SPAD, F_s_ (steady state fluorescence), Fv’/Fm′ (efficiency of PSII in the light-acclimated state), q_p_ (photochemical quenching), qL (fraction of PSII open centers), PhiNO (the portion of energy wasted via non-regulated photosynthesis processes), NPQ (non-photochemical quenching), PhiNPQ (the portion of light wasted due to non-photochemical quenching), and Phi2 (quantum yield of PSII electron transport). Additionally, the impact of the foliar applications of LBS6 on processes related to electron and proton transport such as electron transport rate (ETR_PSII_), proton conductance of chloroplast ATP synthase (gH^+^), electrochomic bandshift (ECSt), change in the proton gradient through thylakoid lumen (vH^+^) representing the rate of ATP production, and photosystem I activity was also evaluated.

#### 2.3.3 Estimation of total soluble sugars and amino acids

The total sugar and amino acid content was measured from the leaves of the corn following the protocol published by Shukla et al. (2023) and Shukla and Prithiviraj (2021), respectively.

#### 2.3.4 Estimation of total phenolics and flavonoids

The leaves from both control and LBS6 treatments, harvested at 35 DAS, were analyzed for total phenolic and flavonoid contents. Initially, 100 mg of leaves was homogenized in liquid nitrogen, and the resulting powder was extracted in 70% methanol and centrifuged at 10,000 rpm for 10 min at 4°C. The extraction process was repeated three times, extracts were pooled, and the volume was adjusted to 15 mL. Total phenolic and flavonoid contents were quantified according to the protocol described by Bajpai et al. (2019).

### 2.4 Determination of soil characteristics, soil enzyme activity, and microbial diversity

#### 2.4.1 Measurement of soil pH, electrical conductivity, and microbial population

The soil samples were prepared as described by Thomas (1996). The pH was measured by a HI 2215 pH/ORP meter (Hanna instruments, United States) and EC was measured by HI 3512 EC and resistivity meter (Hanna instruments, United States). The microbial population was estimated by using the serial dilution and plate count method (Vance et al., 1987). Differential media such as nutrient agar, tryptone soya agar (HiMedia, India), and rose bengal chloramphenicol agar were utilized for counting the colony forming units (CFUs) of total bacteria, actinomycetes, and fungi in the soil samples, respectively.

#### 2.4.2 Determination of soil enzyme activity

For measuring urease activity, soil samples were incubated with urea to extract the ammonium from the soil using 1 N KCl prepared in 0.01 N HCl. Due to urease activity, the released NH^4+^ was measured calorimetrically by a modified indophenol reaction (Kandeler and Gerber, 1988). The activity of aryl sulphatase, acid, and alkaline phosphatase was determined based on the method described by Tabatabai (1982). The soil samples were extracted with modified universal buffer (12.1 g of Tris hydroxymethyl aminomethane, 14 g of citric acid, 11.6 g of maleic acid, 6.3 g boric acid, and 488 mL of 1 N NaOH, and the volume made up to 1,000 mL of water; pH was adjusted using NaOH/HCl) at pH 11 and 6.5 for assaying alkaline and acid phosphatase activity, respectively, with 0.025 M p-nitrophenol phosphate solution as substrate. In total, 4-nitrophenol that released from the reaction due to enzyme activity was quantified calorimetrically using UV–VIS spectrophotometer (NanoQuant, Tecan, Switzerland) at 410 nm. The aryl sulfatase activity was quantified calorimetrically by measuring p-nitrophenol, which is released due to arylsulfatase activity when soil is exposed to a buffered (pH 5.8) solution containing potassium p-nitrophenyl sulfate and toluene and incubated at 37°C for 1 h. Dehydrogenase activity was assayed and was expressed as the rate of formation of triphenyl tetrazolium formazan from 2,3,5-triphenyl tetrazolium chloride as described by Klein et al. (1971). The protocol published by Schnurer and Rosswall (1982) was used for the estimation of fluorescein diacetate (FDA) activity. The reduction in H_2_O_2_ by titration with 0.1 M KMnO_4_ was used to quantify the soil catalase activity (Vijayakumar and Malathi, 2014). The biological index of fertility (BIF) (Stefanic et al., 1984) is calculated as follows:

$\begin{array}{l} BIF=\left(\begin{array}{l} 1.5\;\mathrm{Dehydrogenase}\;\mathrm{activity}+\\ 100\;k\;\mathrm{Catalase}\;\mathrm{activity} \end{array}\right)/2,\\ \mathrm{where}\;k\;\mathrm{was}\;\mathrm{the}\;\mathrm{factor}\;\mathrm{proportionality}\;\mathrm{equal}\;to\;0.01\text{.} \end{array}$

#### 2.4.3 Metagenomic analysis

The influence of foliar application of LBS6 on the rhizosphere microbiome was determined through the amplicon-based next-generation Illumina sequencing of 16S rRNA genes. Bulk soil (BS), SC2, and ST2 (samples collected 7 days after second spray) were used for the analysis as follows.

##### 2.4.3.1 DNA extraction, library preparation, cluster generation, and sequencing

Following the manufacturer’s instructions, metagenomic DNA was extracted from soil samples using a commercially available Nucleospin Soil Kit (Clontech Laboratories, Inc., United States). Nanodrop was used to analyze the quality of the extracted metagenomic DNA sample. The amplicon libraries were constructed using the Nextera XT Index Kit from Illumina Inc., following the 16S metagenomic sequencing library preparation protocol (Part # 15044223 Rev. B). 16S rRNA forward primer (5’-GCCTACGGGNGGCWGCAG-3′) and 16S rRNA reverse primer (5’-ACTACHVGGGTATCTAATCC-3′) required for the amplification of specific regions were designed and synthesized by Eurofins Genomics Lab, Bengaluru, India. The polymerase chain reaction (PCR) was performed to amplify the bacterial 16S region, and 3 μL of the resulting PCR product was separated on 1.2% agarose gel at 120 V for approximately 60 min. Using i5 and i7 primers, which added multiplexing index sequences and common adapters required for cluster generation (P5 and P7), the amplicons with the Illumina adaptors were further amplified in accordance with the standard Illumina protocol. These amplicon libraries were further purified using AMPure XP beads and were quantified using a Qubit Fluorometer. After the quality check of the amplified libraries using 4200 Tape Station System (Agilent Technologies) with D1000 screen tape, libraries were loaded onto the MiSeq platform at proper concentration (10–20 pM), to enable cluster generation and sequencing after the mean peak size from the Tape Station profile was determined. The MiSeq instrument was utilized to perform both forward and reverse sequencing of template fragments through the use of paired-end sequencing. The samples were bound to complementary adapter oligos on the paired-end flow cell using kit reagents. These adapters were created especially to allow the forward strands to be selectively cleaved following the resynthesis of the reverse strand during the sequencing process. The replicated reverse strand was then used to start sequencing from the other end of the fragment.

##### 2.4.3.2 Data analysis

Trimmomatic v 0.38 was used to generate high-quality clean reads by eliminating adapter sequences, vague reads (those with more than 5% unknown nucleotides “N”), and poor-quality sequences (reads with a quality threshold (QV) < 25 phred score in more than 10% of the sequence) using a sliding window of 20 bp and a minimum length of 100 bp. The paired-end (PE) data were merged into single-end reads using FLASH (v1.2.11). The high-quality clean reads that were obtained underwent denoising, and chimeric sequences were removed using the DADA2/Deblur program. Amplicon sequence variants were taxonomically classified using the q2-feature-classifier with a pre-trained classifier based on the SILVA database. To assess diversity, within-sample (α-diversity; Shannon’s index) and between-sample (β-diversity; weighed and unweighted UniFrac) metrics were calculated. The distribution of taxa based on the percentage of operational taxonomic units (OTUs) was visualized in figures that were generated using the QIIME2 program and Microbiome Analyst.^2^

The sequencing data of all 16 samples were submitted in the NCBI Sequence Read Archive under accession No. PRJNA953779.^3^

### 2.5 Statistical analyses

Statistical analysis was carried out using WASP 2.0.^4^ One-way ANNOVA with LSD at a 0.05% probability level was used to analyze the data related to plant morphology, biochemical parameters, soil enzymes, and metagenomics. Tukey’s HSD test (*p* < 0.05) was performed to differentiate the means. Venn diagram was designed using Ugent tool to observe unique and shared species among treatments. Microbiome Analyst (see text footnote 2) was used for bacterial diversity (α diversity and β diversity) and functional analysis. An interactive heatmap was developed in feature-level by using Euclidean distance measure. Correlation analysis was carried out at phylum and class levels using Pearson algorithm with a *p*-value threshold of 0.05. Functional prediction was performed using Tax4Fun function against the SILVA database annotation pipeline and FAPROTAX. The resulting KO table was analyzed for diversity and associations. The R package pheatmap^5^ was used to create the heatmaps.

## 3 Results

### 3.1 LBS6 treatment showed improved morphological growth of *Zea mays*

**Figure 1:**
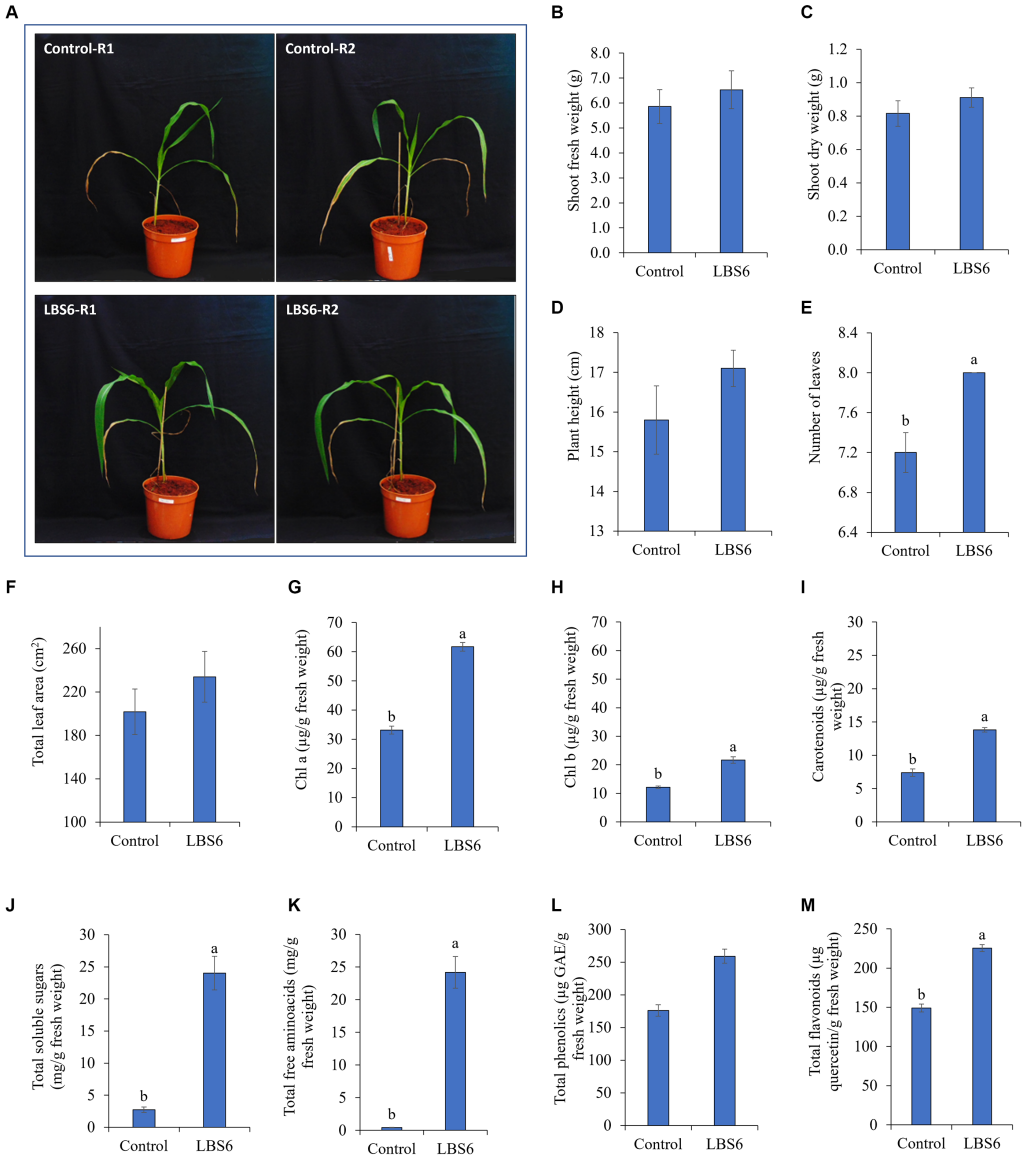
LBS6 improved the growth, pigment content, and biochemical parameters of corn plants. Morphological characteristics **(A–F)**: **(A)** Effect of LBS6 foliar spray on corn plants **(B)** Shoot fresh weight, **(C)** Shoot dry weight, **(D)** Plant height, **(E)** Number of leaves, and **(F)** Total leaf area. Pigment content **(G–I)**: **(G)** Chlorophyll a, **(H)** Chlorophyll b, and **(I)** Total carotenoids. Biochemical characteristics **(J–M)**: **(J)** Total soluble sugars, **(K)** Total free amino acids, **(L)** Total phenolics, and **(M)** Total flavonoids. The values are presented as mean ± SE of ten independent replicates and percentage change compared with control were given with arrow. Significantly different (*p* ≤ 0.05) mean values were represented by different alphabets. The graphs without any letters on the bar were not statistically different.

The plants treated with LBS6 (1 mL. L^−1^) showed higher growth than the control plants (Figure 1A). LBS6-treated plants showed 11 and 12% higher fresh and dry weight of shoot, respectively (Figures 1B,C), as compared with the control. Similarly, plant height was also observed to be increased by 8% in LBS6-sprayed plants (Figure 1D). The plants sprayed with 1 mL/L of LBS6 also showed a significantly higher number of leaves than the control (Figure 1E). Foliar spray of LBS6 also had a positive impact on average leaf area, which was increased by 16% in treated plants as compared with the control; however, the values are not significantly different (Figure 1F).

### 3.2 Effect of LBS6 treatment on pigment content of *Zea mays*

Chlorophyll A and B contents were found significantly higher in corn plants sprayed with 1 mL. L^−1^ of LBS6 (Figures 1G,H). When compared with the control, it was found that the levels of chlorophyll a and b had significantly increased by 86 and 79%, respectively. Total carotenoid content of LBS6-sprayed plants was also found to be significantly increased by 87%, as compared with the control (Figure 1I).

### 3.3 Effect of foliar spray of LBS6 on total soluble sugar, amino acid, phenolic, and flavonoid contents of leaves of *Zea mays*

The total soluble sugar content was notably higher (9-fold) in plants treated with LBS6, as compared with the control (Figure 1J). Total amino acid content was also significantly increased by 60-fold in plants sprayed with LBS6, as compared with the control plants (Figure 1K). Total phenolics and flavonoids involved in inducing resistance against abiotic stress, were found to be increased with foliar spray of LBS6. Total phenolic content was significantly increased by 47% in LBS6-treated plants, as compared with the control (Figure 1L). Total flavonoids, measured as quercetin equivalents, were significantly increased by 51% with LBS6 treatments, as compared with the control (Figure 1M).

### 3.4 Effect of LBS6 on chlorophyll fluorescence and photosynthesis-related parameters

Soil Plant Analyzer Development (SPAD) values represent relative chlorophyll content and were observed to be higher in LBS6-treated plants than in control (Table 1); however, the values were not statistically significant. Additionally, leaf thickness of LBS6-sprayed plants was significantly higher as compared with control plants. PS1 active and oxidized centers were higher, while PS1 reduced and open centers were less in LBS6-treated plants; however, the changes were not statistically significant (Table 1). Steady state fluorescence (F_s_) was less in those LBS6-treated plants, as compared with the control, but the values were not statistically different. The efficiency of the PSII in the light-accumulated state (F_v_^’^ over F_m_^′^) and the fraction of PSII open centers (qL) were observed to be increased by a foliar application of LBS6, as compared with the control. The quantum yield of photosystem II (Phi2) was higher in LBS6-treated plants than in control, which was considered indicative of more conversion of light energy to photochemical reactions in PSII. Non-photochemical quenching (NPQ_t_) and the portion of incident light dissipated as heat energy (PhiNPQ) were observed to be less in the treated plants than in the control (Table 1). The changes monitored in the portion of incident light lost via non-regulated processes (PhiNO) in both control and LBS6-sprayed plants were not statistically significant. These results indicated that the foliar application of LBS6 increased the efficiency of photosynthesis-related processes in plants. Higher rates of the total flow of electrons from antenna complexes into PSII (LEF), the rate of electrochromic shift (ECS_tau), and steady-state rate of proton flux through the chloroplast ATP synthase (gH^+^) were observed in LBS6-sprayed plants as compared with the control plant. In comparison to the control, it was found that the plants sprayed with LBS6 showed higher rate of linear electron transport, which is determined by the portion of the light used for photosynthetic processes in PSII under the light-adapted state (ETR_PSII_).

**Table 1:** Effect of LBS6 foliar spray on chlorophyll fluorescence, electron, and proton transport-related processes in light from leaves of the corn plants.

Parameters	^Control^	^LBS6^
ECS_tau	0.008 ± 0^a^	0.009 ± 0^a^
Fs	866.844 ± 14.67^a^	848.079 ± 14.8^a^
FvP_over_FmP	0.684 ± 0.01^a^	0.696 ± 0.01^a^
gH+	128.448 ± 8.82^a^	135.147 ± 15.34^a^
aleaf_thickness	0.228 ± 0.05^b^	0.266 ± 0.04^a^
LEF	29.945 ± 2.65^a^	35.248 ± 4.07^a^
Light intensity (PAR)	113.712 ± 9.93^a^	131.319 ± 15.18^a^
NPQt	1.298 ± 0.14^a^	1.15 ± 0.09^a^
Phi2	0.586 ± 0.01^a^	0.604 ± 0.01^a^
PhiNO	0.184 ± 0^a^	0.186 ± 0^a^
PhiNPQ	0.23 ± 0.02^a^	0.21 ± 0.01^a^
PS1 active centers	0.998 ± 0.12^a^	1.068 ± 0.05^a^
PS1 open centers	0.27 ± 0.13^a^	0.181 ± 0.02^a^
PS1 over reduced centers	0.612 ± 0.13^a^	0.575 ± 0.03^a^
PS1 oxidized centers	0.118 ± 0.13^a^	0.244 ± 0.02^a^
qL	0.652 ± 0.01^a^	0.667 ± 0.01^a^
SPAD	30.923 ± 0.72^a^	31.56 ± 1.04^a^
vH+	0.046 ± 0^a^	0.045 ± 0^a^
ETR_PSII_	27.948 ± 2.47^a^	32.899 ± 3.8^a^

Note: The values were presented as mean±SE, and the significantly different (p ≤ 0.05) mean values were represented by different letters.

### 3.5 Foliar applications of LBS6 improved physiochemical properties, microbial count, and enzyme activity of soil grown with *Zea mays*

#### 3.5.1 Soil physiochemical property and microbial count

The foliar spray of LBS6-modulated physiochemical properties and microbial count of soil (Table 2). Nutrient availability to the plants is affected by pH of the soil as it is one of the main factors that regulate solubility of nutrients in soil water. A reduction in pH of the rhizospheric soils was observed during plant growth. The pH of rhizospheric soil collected 7 days after the second foliar spray of LBS6 (ST2) was observed to be 6.9, while the pH of the rhizospheric soil from the roots of control plants (SC2) at the same time-point was found to be 6.7. Electrical conductivity (Ec) measures the ion exchange capacity of soils and gives an indirect assessment related to the quantity of available soil nutrients. Electrical conductivity was significantly reduced by 43 and 62% in rhizospheric soil ST1 and ST2 collected from roots of LBS6-sprayed plants as compared with rhizospheric soil SC1 and SC2 collected from control plants after third and seventh day of the second spray, respectively. The soil samples showed an increase in CFU/g of bacteria and fungi during plant growth. The total bacterial count was increased by 57% (ST1) and 52% (ST2) in soil samples collected from LBS6-sprayed plants as compared with soil samples collected from control plants, respectively. It is interesting to note that, following the second foliar spray, the fungal population in ST2 increased by 54% over ST1, while there was no change in the fungal population in the control samples at either time-point. The population of actinomycetes was higher in rhizospheric soil collected from control plants than in the rhizospheric soil from LBS6-sprayed plants.

**Table 2:** Effect of the foliar application of LBS6 on pH, EC, and number of bacteria, fungi, and actinomycetes in soil samples.

Treatments		pH	Ec (μS/cm)	Total bacterial population in soil (CFU/g of soil)	Total fungal population (CFU/g of soil)	Total actinomycetes population (CFU/g of soil) ***
BS*	Control	7.37	186.1	0.05 × 10^5^	0.29 × 10^2^	0.59 × 10^4^
3 DAD2**	Control	7.09	222.70	1.90 × 10^5^	2.84 × 10^2^	1.00 × 10^4^
	LBS06	7.08	156.10	4.44 × 10^5^	1.83 × 10^2^	0.76 × 10^4^
7 DAD2	Control	6.72	275.10	0.52 × 10^5^	2.84 × 10^2^	3.65 × 10^4^
	LBS06	6.88	169.70	1.08 × 10^5^	3.95 × 10^2^	0.49 × 10^4^

The values were presented as mean ± SE, and means represented by the same letters in superscript were not significantly different at *p* ≤ 0.01. *BS—Bulk soil sample, collected from unplanted soil. **DAD2—days after treatment dose 2 application. ***CFU—colony forming units.

#### 3.5.2 Foliar application of LBS6 differentially regulates the activity of soil enzymes

**Figure 2:**
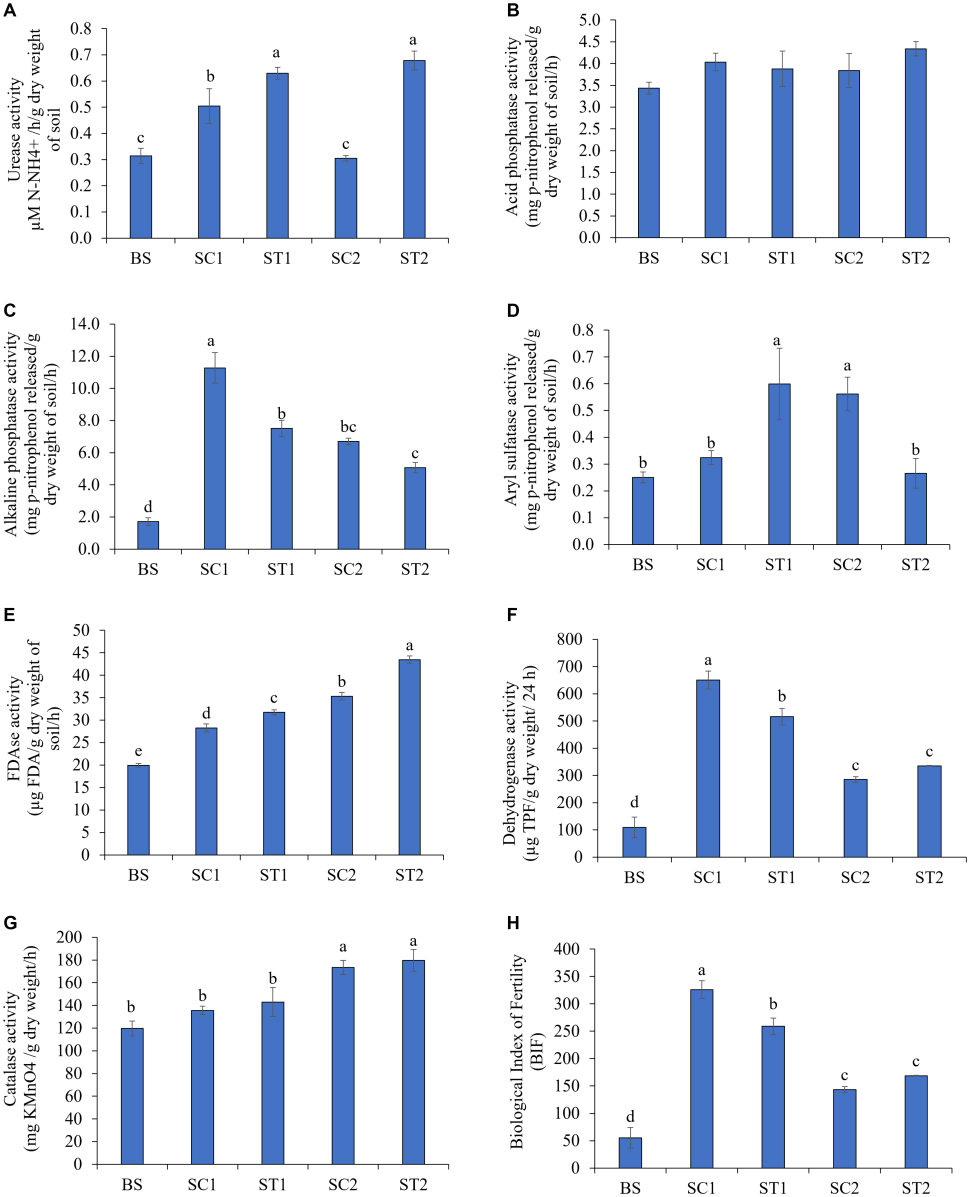
LBS6 regulated the activity of soil enzymes involved in nutrient cycles representing soil microbial activity. Nutrient cycle **(A-D)**: **(A)** Urease, **(B)** Aryl sulphatase, **(C)** Acid phosphatase, and **(D)** Alkaline phosphatase. Microbial activity **(E-H)**: **(E)** FDAse, **(F)** Dehydrogenase, **(G)** Catalase, and **(H)** biological index of fertility. BS—Bulk soil; SC1 and SC2—rhizosphere soil samples collected from control plants sprayed with water on 3rd and 7th days after second spray of water. ST1 and ST2—soil collected 3 and 7 days after second spray of LBS6. The values are presented as mean ± SE of three independent replicates, and significantly different mean values (*p* ≤ 0.05) were represented by different letters. The graphs without any letters on the bar were not statistically different.

The urease used in urea degradation in treated soils is considered to be a good proxy for nitrogen availability in the soils of treated plants. The urease activity was found to be increased in rhizospheric soils during plant growth, as compared with bulk soil. Rhizospheric soils collected from LBS6-sprayed plants (ST1 and ST2) showed 20 and 55% enhanced activity of urease enzyme, respectively, as compared with soil collected from roots of the control plants (SC1 and SC2) (Figure 2A). The increment of urease activity in soil samples from treated plants was statistically significant, as compared with the control samples. Acid and alkaline phosphatases catalyze the phosphate bond cleavage to convert unavailable phosphate into available form and act as an indicator for soil phosphorous mineralization (Krämer and Green, 2000). Rhizospheric soil from LBS6-sprayed plants collected 7 days after second spray (ST2) showed a 12% increase in activity of acid phosphatase, as compared with the soil collected from the control plants at the same time-point (SC2), but the changes are not significantly different (Figure 2B). The activity of alkaline phosphatase measured as mg p-nitrophenol released/g dry weight/h was 11.2 in the SC1, which was reduced to 6.7 in SC2; however, the activity of the same enzyme was 7.5 in soil samples collected after 3 days of LBS6 spray (ST1), which was reduced to 5.1 in soil samples collected after 7 days of LBS6 spray (ST1) (Figure 2C). Aryl sulfatase, an important enzyme involved in sulfur cycling in soil, was involved in the acquisition of organic sulfur. Its activity was increased in rhizospheric soils collected on third day after second spray of LBS6 (ST1) but was found to be reduced in soil samples collected after seventh day of second spray of LBS6 (ST2) (Figure 2D). ST1 samples had 46% increase in aryl sulfatase activity than SC1 samples.

FDAse activity measured the overall microbial population and soil quality and was significantly increased by 11 and 19% in rhizospheric soil collected from plants sprayed with LBS6 (ST1 and ST2), as compared with soil from control plants (SC1 and SC2), respectively (Figure 2E). After 7 days of second foliar spray, dehydrogenase activity was observed to be 15% higher in soil collected from LBS6-treated plants (ST2), as compared with the soil collected from control plants at the same time-point (SC2) (Figure 2F). Catalase activity is typically characterized by the highest content of organic matter creating favorable conditions for microorganisms. Rhizospheric soil from the LBS6-sprayed plants (ST1 and ST2) showed 5 and 3% higher catalase activity, respectively, compared to the soil from control plants (SC1 and SC2), but the change was not statistically significant (Figure 2G). After 3 days of spray, biological index of fertility (BIF) was found to be increased in soil samples collected from both control (SC1) and LBS6 (ST1)-treated plants. With the progression of time, BIF was reduced in both SC2 and ST2, but the reduction was less in ST2 than in SC2 (Figure 2H). After 7 days of second spray of LBS6 (ST2), the collected soil showed an increase of 15% in BIF values as compared with control SC2. These findings conclude that the foliar spray of LBS6 improves the microbial population of the rhizospheric soil.

#### 3.5.3 Effect of the application of LBS6 on soil microbial diversity by metagenome analysis and functional prediction

In the metagenomics study, 14,95,467 high-quality effective reads were obtained from nine samples, with an average of 1,66,163, ranges from 1,39,564 to 1,94,851 reads/sample. After clustering with 97% identity, chimeric sequences were removed to generate 8,018 operational taxonomic units (OTUs). These bacterial OTUs were grouped into 35 phyla, 110 classes, 268 orders, 421 families, 791 genera, and 1,381 species.

##### 3.5.3.1 Rarefaction curve and alpha and beta diversity of the bacterial population

The construction of rarefaction curves represents intra-diversity bacterial richness, and those samples whose rarefaction curve reached near the plateau showed that the sampling depth and sequence coverage were satisfactory for the given set of samples (Supplementary Figure S2A). Species richness was higher in rhizospheric soil during plant growth and was similar in soil collected from the rhizosphere of plants after 7 days of second foliar spray of water (SC2) and LBS6 (ST2). However, the number of sequences was higher in rhizospheric soil from the plants sprayed with LBS6 (ST2). Alpha diversity of the samples was calculated from Chao1, Shannon, and Simpson indices at the phylum level. Chao1 index values revealed that SC2 and ST2 had the highest species richness with more rare species, as compared with the bulk soil (Supplementary Figure S2B). However, a slight reduction in the Chao1 value was observed in ST2 samples, as compared with SC2. The Shannon index which estimates both the species richness and evenness was higher in both rhizospheric soil collected from plants sprayed with LBS6 (ST2) and water (SC2), as compared with the bulk soil. However, the species richness and evenness were slightly less in ST2, as compared with SC2 (Supplementary Figure S2C). The Simpson index measured the diversity, with the relative abundance of each species, Supplementary Figure S2D was statistically similar in ST2 and SC2 samples. The bacterial community diversity, also termed as beta diversity based on genera calculated by principal coordinate analysis using the Bray–Curtis index, is shown in Supplementary Figure S2E. The Bray–Curtis index values showed how different bacterial communities were in different rhizospheric soil samples (BS, SC2, and ST2). Based on the Bray–Curtis index values, the microbial communities in the base soil were different from that of SC2 and ST2, suggesting that plants recruit more rhizospheric microbial communities during its growth. The principal component analysis revealed that replicates of samples collected from LBS6-sprayed plants (ST2) were clustered together, while SC2 sample replicates were scattered and loosely grouped (Supplementary Figure S2E).

##### 3.5.3.2 Impact of foliar applications of LBS6 on the distribution of bacterial communities in the soil grown with *Zea mays*

**Figure 3:**
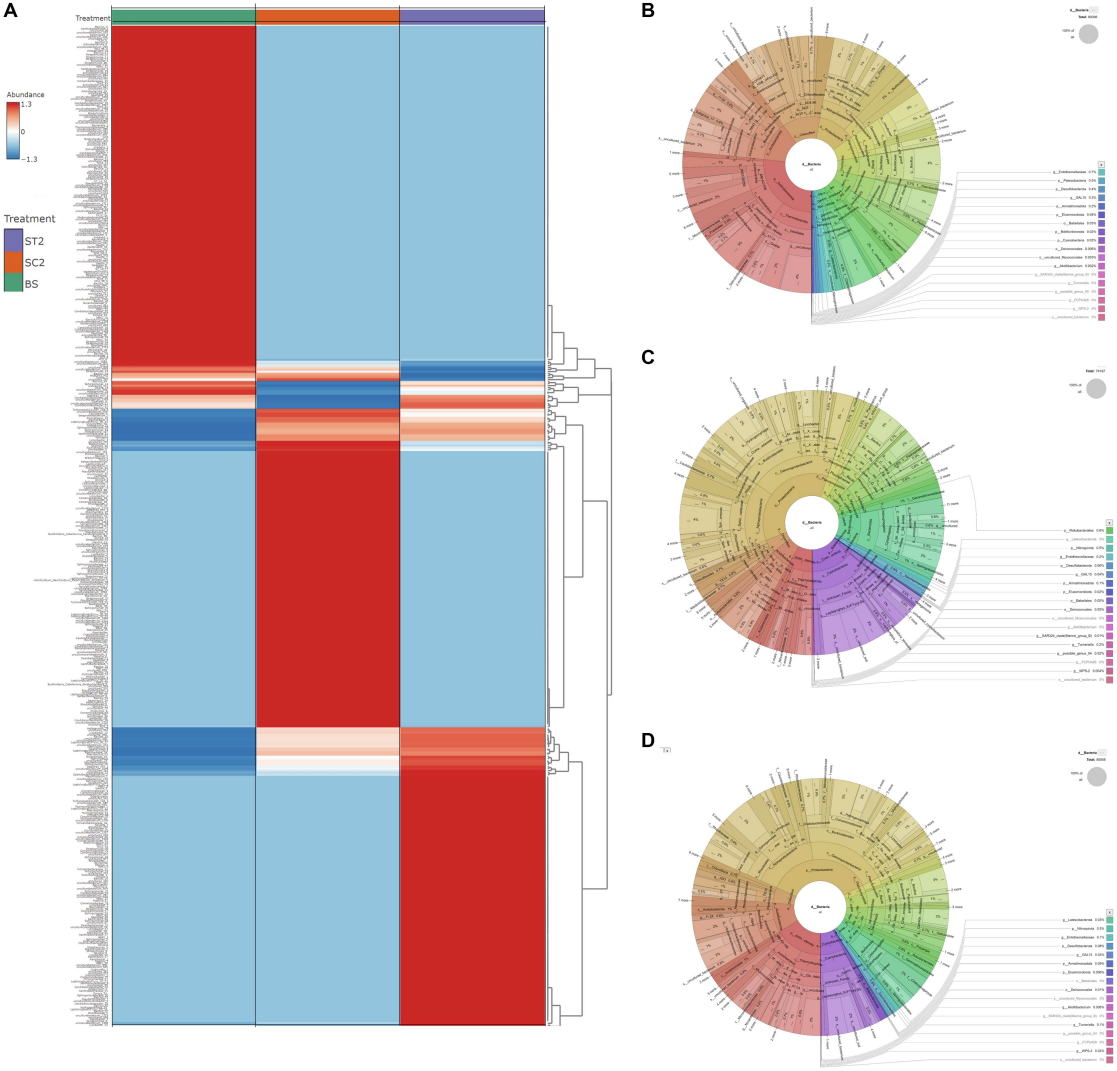
Difference in bacterial diversity between soil samples. **(A)** Bacterial composition for BS, SC2, and ST2 sample groups at the genus level. **(B–D)** Charts show the diversity of kingdom bacteria in soil samples. **(A)** BS, **(B)** SC2, and **(C)** ST2 (classified by SILVAngs and displayed in KRONA). BS—Bulk soil; SC2—rhizosphere soil samples collected after 7 days of second spray of water. ST2—soil collected 7 days after second spray of LBS6.

**Figure 4:**
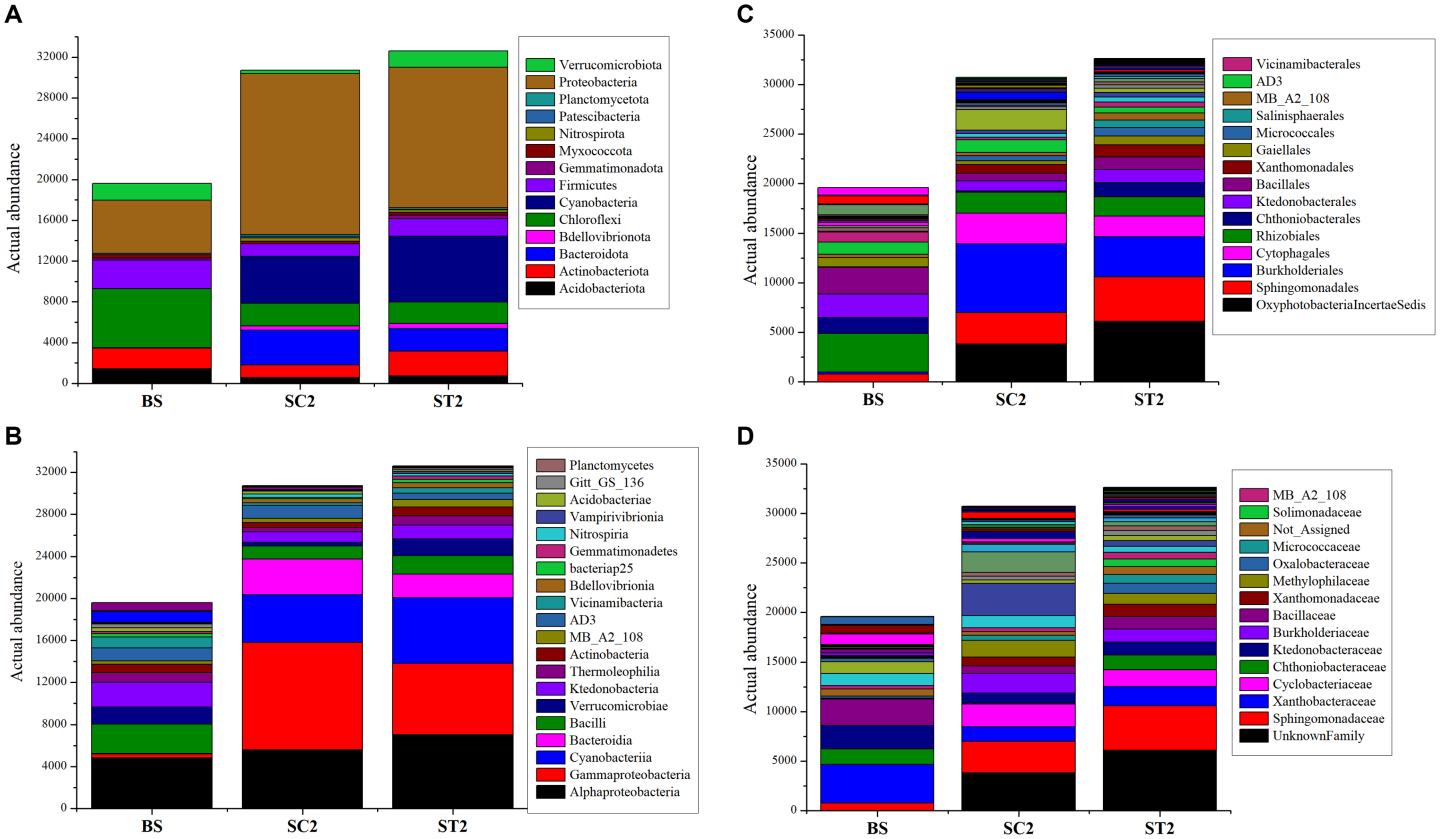
Charts showing distribution of taxa based on actual abundance. **(A–D)** Charts represent the taxa at phylum, class, order, and family level. The top taxa were observed here and the sequences that did not have any alignment against the taxonomic database were categorized as “not assigned”.

The taxonomy annotation and abundance of bacterial species obtained from the three sample groups were analyzed based on the predicted OTUs. Bacterial composition for BS, SC2, and ST2 sample groups at the genus level is shown in Figure 3A. Enrichment of bacterial communities was clearly different among the three sample groups. The dominating phyla in soil samples were Proteobacteria, Cyanobacteria, Chloroflexi, Firmicutes, Actinobacteriota, Bacteroidota, Verrucomicrobiota, Acidobacteriota, Myxococcota, Bdellovibrionota, Nitrospirota, and Gemmatimonadota, accounting for more than 90% of total bacteria (Figures 3B–D; Supplementary Figure S3A). Bulk soil showed less bacterial population, and it is observed that the growing plants improve the soil bacterial diversity (SC2 and ST2) (Figure 4A). The foliar spray of *Kappaphycus*-derived biostimulants (LBS6) had a significant impact on the distribution of different rhizospheric bacterial communities. The rhizospheric soil samples from the LBS6-sprayed plants (ST2) showed 6% increase in the actual abundance of bacteria as compared with those present in the control rhizospheric soil (SC2). Bulk soil was abundant in the following phyla: Chloroflexi, Proteobacteria, Firmicutes, Actinobacteriota, Verrucomicrobiota, and Acidobacteriota (Figure 4A) The relative abundance of Proteobacteria, Cyanobacteria, Bacteroidota, Bedellovibrionota, Nitrospirota, and Patescibacteria was increased, and the relative abundance of Chloroflexi, Firmicutes, and Acidobacteriota was decreased in the rhizospheric region of soil during plant growth in both control and LBS6-sprayed plants. The rhizospheric soil samples collected from plants sprayed with LBS6 (ST2) showed an increase in the relative abundance of Cyanobacteria, Actinobacteriota, Firmicutes, Verrucomicrobiota, Acidobacteriota, Bdellovibrionota, Myxococcota, Gemmatimonadota, and Planctomycetota by 29, 49, 29, 78, 23, 22, 18, and 13%, respectively, while decreases in Bacteroidota and Proteobacteria by 53 and 14%, respectively, were observed, as compared with the rhizospheric soil collected from control plants (SC2).

All rhizospheric soil samples were dominated by classes Alphaproteobacteria, Gammaproteobacteria, Cyanobacteria, Bacilli, Bacteroidia, Ktedonobacteria, and Actinobacteria (Supplementary Figure S3B). Alphaproteobacteria, Bacilli, Ktedonobacteria, and Actinobacteria were most abundantly present in bulk soil (Figure 4B). SC2 was dominated by Gammaproteobacteria (33%), Alphaproteobacteria (18%), Cyanobacteria (15%), Bacteroidia (11%), and *Bacilli* (4%), while ST2 was dominated by Alphaproteobacteria (22%), Gammaproteobacteria (21%), Cyanobacteria (19%), Bacteroidia (7%), and Bacilli (5%). Increase in the abundance of the classes Alphaproteobacteria, Cyanobacteria, Bacilli, Verrucomicrobiae, Thermoleophillia, Actinobacteria, and Vicinamibacteria by 20, 28, 29, 54, 45, and 52%, respectively, were observed in ST2 compared with SC2. Gammaproteobacteria and Bacteroidia were reduced by 50 and 53%, respectively, in the soil samples collected from the plant sprayed with LBS6 (ST2) as compared with control plants (SC2). The rhizospheric soil from plants sprayed with LBS6 (ST2) was mainly enriched with the orders Azospirillales, Bacillales, Acidobacteriales, Caeenarcaniphilales, Micrococcales, Oxyphotobacteria, Paenibacillales, Pseudomonadales, and Sphingomonadales by 52, 100, 39, 100, 45, 37, 21, 17, and 29%, respectively, as compared with control plants sprayed with water (Figure 4C; Supplementary Figure S3C). Some notable bacterial families Micrococcaceae, Bacillaceae, Sphingomonadaceae, and Paenibacillaceae were increased by 45, 39, 29, and 21%, respectively, in ST2 compared with SC2 (Figure 4D).

**Figure 5:**
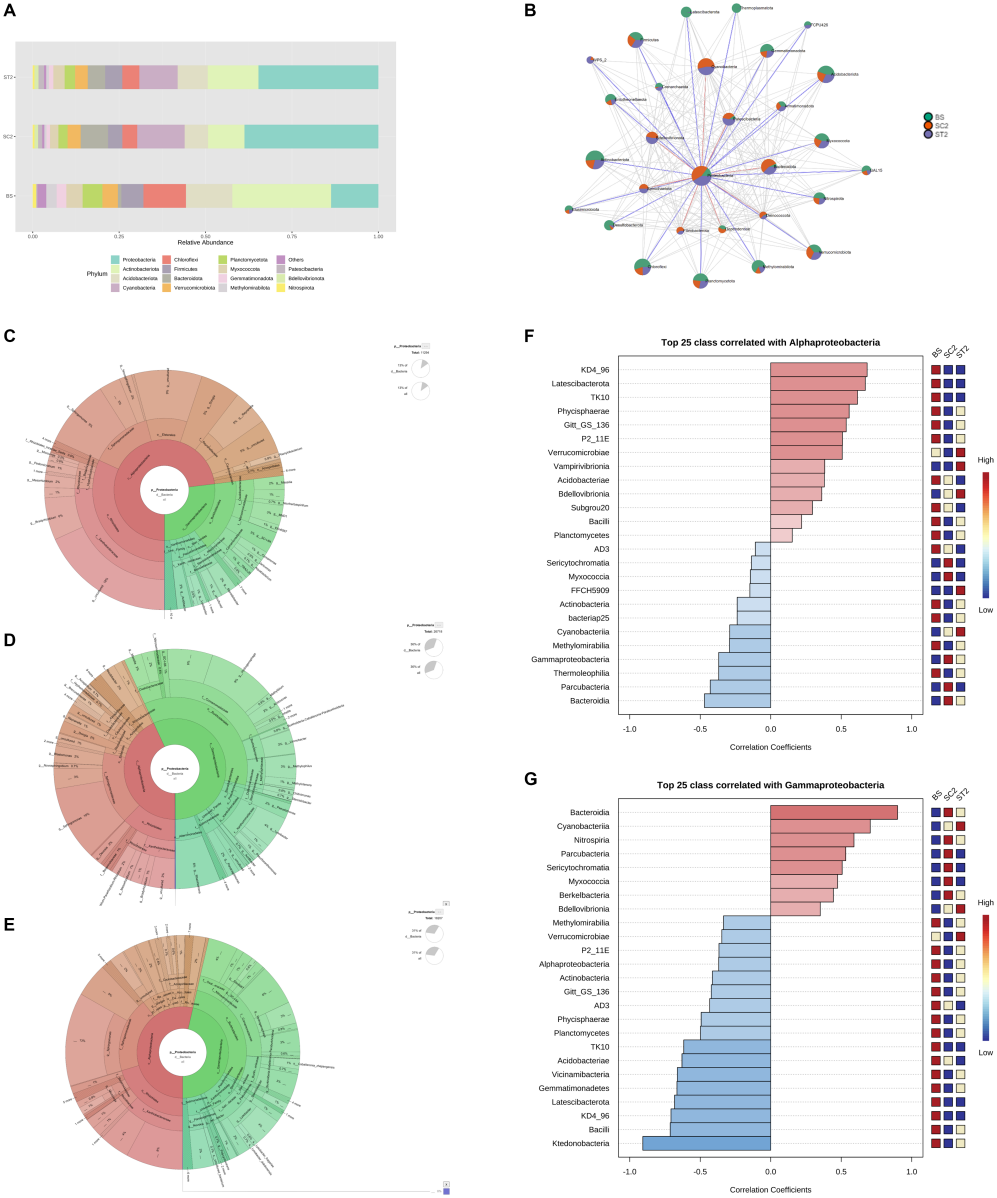
Differences in the diversity of phylum Proteobacteria. **(A)** Bacterial relative abundance at phylum level. **(B)** Correlation of Proteobacteria with other phyla. Differences in the diversity of Proteobacteria between samples were shown in charts **(C–E)**. **(C)** BS, **(D)** SC2, and **(E)** ST2 (Classified by SILVAngs and displayed in KRONA). Correlation of classes Alphaproteobacteria **(F)** and Gammaproteobacteria **(G)** with other classes were shown. BS—Bulk soil; SC2—rhizosphere soil samples collected after 7 days of second spray of water. ST2—soil collected 7 days after second spray of LBS6.

The rhizospheric soil samples grown with water-sprayed plants (SC2) were dominated by members of the phylum Proteobacteria, which accounted for 47% of the total bacterial community, followed by 15.8% Cyanobacteria and 11.8% Bacteroidota, while ST2 samples had 42% Proteobacteria, 16.4% Cyanobacteria, and 8.7% Actinobacteria (Figure 5A). The increase in Proteobacteria was positively associated with the abundance of Actinobacteriota, Acidobacteriota, Firmicutes, and Verrucomicrobiota and negatively correlated with Cyanobacteria and Bacteroidota in both soil samples grown with plants sprayed with water (SC2) and LBS6 (ST2) (Figure 5B). This suggested that plant growth improved the enrichment of Proteobacteria in soil. Interestingly, the relative abundance of phlya Actinobacteriota, Firmicutes, Chloroflexi, Acidobacteriota, and Verrucomicrobiota was found to be higher in ST2 samples as compared with SC2 samples (Figure 5A).

The differences in members of the phylum Proteobacteria between samples are shown in Figures 5C–E. Bulk soil was dominant in the class Alphaproteobacteria. Rhizosphere soil collected from LBS6-sprayed plants (ST2) maintained the population of both Alphaproteobacteria (48.7%) and Gammaproteobacteria (51.3%) equally, while Gammaproteobacteria (61%) was dominated in rhizospheric soil collected from control plants (SC2). Alphaproteobacteria which was dominant in ST2 was positively correlated with Verrucomicrobia, Bacilli, and Latescibacterota (Figure 5F). Gammaproteobacteria which was dominant in SC2 was positively correlated with the classes Bacteroidia, Cyanobacteria, and Nitrospiria (Figure 5G). However, Gammaproteobacteria was negatively correlated with most of the classes with plant beneficial bacteria such as Alphaproteobacteria, Actinobacteria, Verrucomicrobiae, Bacilli, and Latescibacterota.

Venn diagrams were created to show how many distinct and common genera, species, and OTUs were found in each sample (Supplementary Figure S4). The total number of different genera identified in bulk soil, SC2, and ST2 was 70, 65, and 69, respectively (Supplementary Figure S4A). Bulk soil, SC2, and ST2 have 235, 209, and 224 different species, respectively (Supplementary Figure S4B). The number of different OTUs present in bulk soil has 2,687, in SC2 has 2,535, and in ST2 has 2,674 (Supplementary Figure S4C). The number of genera, species, and OTUs shared by all samples were 223, 357, and 8, respectively. The number of genera, species, and OTUs shared between SC2 and ST2 is 90, 196, and 70, respectively. Bulk soil and SC2 shared 28 genera, 69 species, and 15 OTUs. A total of 31 genera, 91 species, and 29 OTUs were shared between bulk soil and ST2. SC2 and ST2 had the highest number of shared genera, species, and OTUs, which indicates the similar changes in bacterial community during plant growth. The number of shared genera, species, and OTUs was higher between bulk-soil and ST2 compared with that of between bulk soil and SC2. The number of different genera, species, and OTUs was also higher in ST2 compared with SC2. To conclude, ST2 managed to retain initial soil bacterial communities and had higher distinctive rare taxa as compared with SC2.

##### 3.5.3.3 Foliar spray of LBS6 modulated the bacterial community-associated functions in the soil grown with *Zea mays*

**Figure 6:**
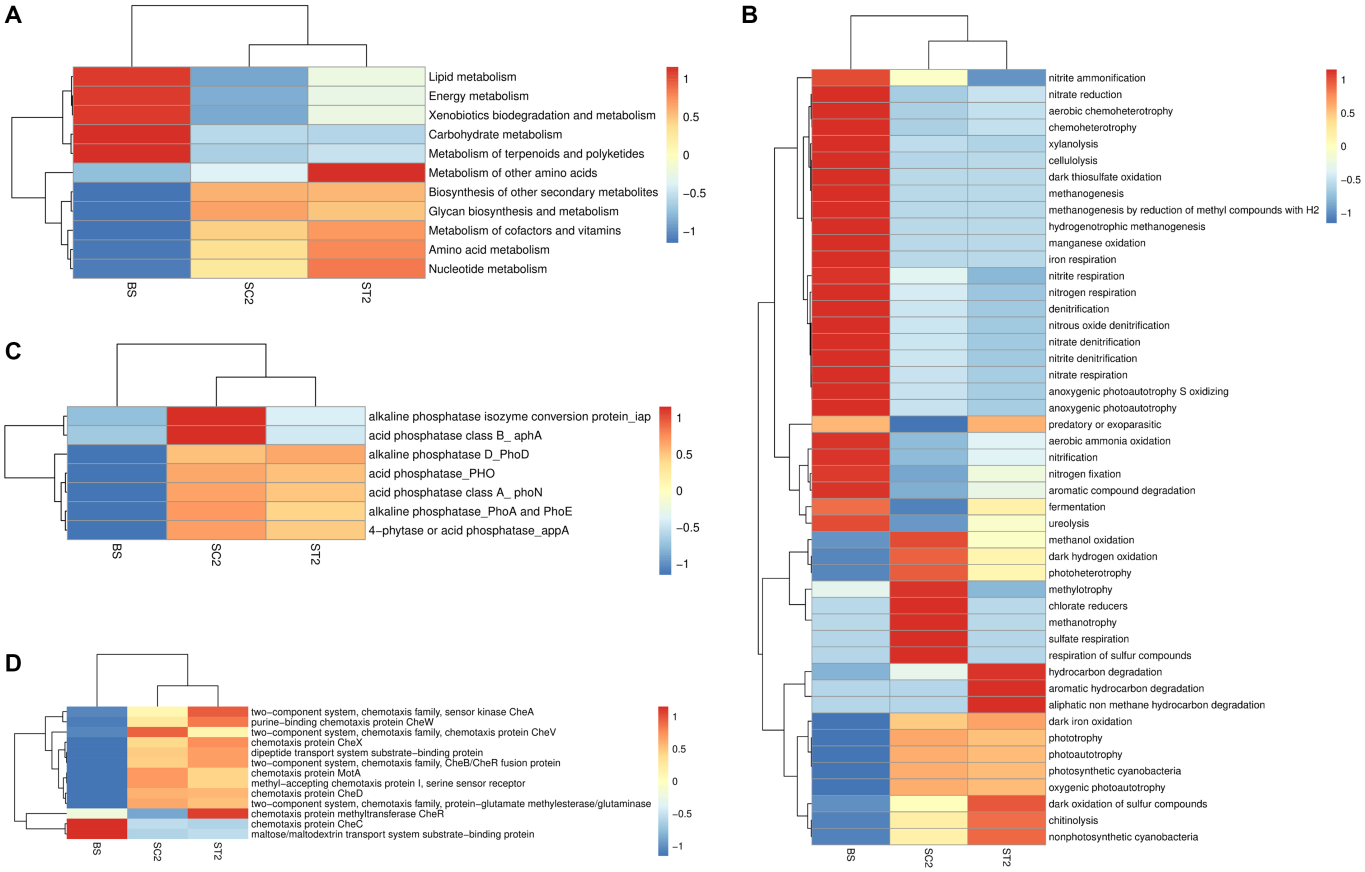
LBS6 spray induced differences in the functional profile obtained based on soil microorganisms (Tax4Fun and FAPROTAX analysis). **(A)** Difference in the KEGG metabolisms, **(B)** nitrogen and sulfur metabolism, **(C)** phosphorous metabolism, and **(D)** bacterial chemotaxis. BS—Bulk soil; SC2—rhizosphere soil samples collected after 7 days of second spray of water. ST2—soil collected 7 days after second spray of LBS6.

Tax4Fun and FAPROTAX analyses were carried out to compare the differences between metabolic functions of bacterial communities present in the rhizospheric soil of LBS6-treated plants and those collected from the roots of control plants (Figure 6). QIIME was used against the SILVA database input by Tax4Fun, to obtain the KO (KEGG Ortholog) table. The obtained KO table data were filtered to remove low-abundance features, based on prevalence, and assess the variations between functional profiles of samples. Heatmaps that are generated from KO table using Euclidean distance measure showed the differences in metabolic profiles of rhizospheric soil samples (Supplementary Figure S6A). A clear difference in metabolic functions was observed between different soil samples (BS, SC2, and ST2,). Nearly 80% of the KEGG orthologs showed high abundance in ST2 compared with SC2 and bulk soil. Enrichment in lipid, energy, amino acid, co-factor, and vitamin synthesis, and nucleotide metabolism was observed with LBS6 treatment (Figure 6A).

In FAPROTAX analysis, rhizospheric soil collected from LBS6-sprayed plants showed that enrichment of pathways was related to nitrogen metabolism (Figure 6B). Functions such as nitrogen fixation, nitrate reduction, aerobic ammonia oxidation, and nitrification were increased in ST2 by 18, 20, 88, and 88% respectively, as compared with SC2. The denitrification, a process that converts available nitrogen into a gaseous form, was reduced in ST2 by 29%, as compared with SC2. Nitrogen, nitrate, and nitrite respiration was also reduced in ST2 by 35, 43, and 42%, respectively, as compared with SC2. The foliar spray of LBS6 modulated the rhizospheric bacterial functions involved in sulfur cycle. Sulfate respiration and respiration of other sulfur compounds and anoxygenic sulfur oxidization were found to be higher in rhizospheric soil collected from the control plants (SC2) than in the soil collected from the LBS6-sprayed plants (ST2), while oxidation of sulfur compounds was found to be higher in ST2, as compared with SC2. Processes and bacterial communities related to carbon fixation were enriched in rhizosphere soil during the plant growth. ST2 also showed increased metabolic functions related to aromatic and aliphatic compound degradations and cellulolytic and chitinolytic activity as compared with SC2 (Figure 6B).

Bacteria involved in phosphatase activity were increased during plant growth and were differentially regulated in SC2 and ST2 samples (Figure 6C). Bacteria-based alkaline phosphatase D (PhoD) was found to be increased in ST2 as compared with SC2, while microbial functions related to phosphorous solubilization were enriched in SC2. The functions related to bacterial chemotaxis were observed to be increased in soil samples collected from LBS6-sprayed and control plants as compared with the bulk soil. Chemotaxis protein CheR and CheX and two component system sensor kinase CheA and CheB/CheR fusion proteins were increased in ST2 compared with SC2 (Figure 6D). These results provided evidence that LBS6-sprayed plants modulate rhizospheric soil bacterial diversity and bacteria-dependent soil functions.

## 4 Discussion

Conventional intensive agricultural practices depend on the excessive application of chemical fertilizers and pesticides, leading to worldwide environmental degradation (Tang et al., 2022). Most adversely affected are soil ecosystems, and this leads to many issues such as lowering soil fertility by impacting the soil microbial community, abundance, and diversity and thus negatively impacts the plant health (Spångberg et al., 2014). There is an urgency for innovating a sustainable environmentally friendly alternative to reduce, if not replaced, the use of these synthetic chemicals in the agricultural practices (Chaparro et al., 2012). Plant biostimulants are classes of the agricultural inputs that are known to improve agricultural yield by improving nutrient use efficiency and mitigating abiotic stress (du Jardin, 2015; Van Oosten et al., 2017; Yakhin et al., 2017; Shukla et al., 2019, 2022; Muhie, 2023). Among different classes of plant biostimulants, seaweed-based biostimulants (phycobiostimulants), as differentiated from acids (humates and aminos), protein hydrolysates and botanical extracts, are the most studied for their functionalities and offer clear alternatives to chemical inputs for higher agricultural yield in a sustainable manner (Shukla et al., 2019). Sap derived from commercially cultivated *Kappaphycus alvarezii* has been researched to improve the yield of various crops, such as rice, potato, and corn (Mondal et al., 2015; Pramanick et al., 2017; Layek et al., 2018). Improvement due to the application of *K. alvarezii* sap on various morphological characteristics such as leaf area, plant height, dry matter, root length, volume, and lateral roots was reported in corn (Mondal et al., 2015; Singh et al., 2016). Yield parameters such as seed yield, cob length, and number of cobs were also found to be improved in corn due to the application of *Kappaphycus* extracts (Trivedi et al., 2017, 2018; Kumar et al., 2020).

In this study, the effects of foliar applications of more differentiated *K. alvarezii*-derived biostimulant (LBS6) at V3 and V7 stages on the morphological, photosynthetic, and biochemical characteristics on the early stages of growth were evaluated. Furthermore, the effect of the application of *Kappaphycus*-derived biostimulant (LBS6) at early stages of vegetative growth and photosynthetic and biochemical characteristics was studied as being important for microbial colonization of the rhizosphere.

### 4.1 Foliar spray of LBS6 enhanced the growth of *Zea mays* by modulating photosynthesis and metabolic-related processes

The foliar spray of LBS6 at V3 and V7 stages of corn improves the growth of plants (Figure 1). The plants sprayed with LBS6 had a larger leaf area and number of leaves with increased chlorophyll and carotenoid contents as compared with the control plants. The LBS6-treated plants were taller than control plants. Fresh and dried biomass of the shoot was also found higher in LBS6-sprayed plants. Various scientific findings showed that the supplementation of *K. alvarezii*-extracted sap improved the morphological and physiological parameters of corn plants (Mondal et al., 2015; Singh et al., 2016; Trivedi et al., 2017, 2018; Kumar et al., 2020). Singh et al. (2016) also showed an increased leaf area in corn with the application of *Kappaphycus* extract. The foliar application of LBS6, an extract from *K. al*var*ezii*, showed more growth of leaves by regulating the processes involved in cell growth, cell proliferation, cell expansion, and phytohormone metabolism (Shukla et al., 2023). SPAD values indicated relative chlorophyll content (Kandel, 2020) and were found to be increased in LBS6-treated plants as compared with the control. The chlorophyll index was also found to be higher in corn with the application of *Kappaphycus*-derived sap (Mondal et al., 2015). Similarly, Shukla et al. (2023) also reported significantly higher SPAD values in cucumber leaves sprayed with LBS6. The SPAD values reported a higher chlorophyll content in LBS6-sprayed leaves, suggesting an enhanced capacity for the absorption of light energy for photosynthesis. Light interception capacity of the crop was determined by the leaf growth. Increased leaf area indicate enhanced photosynthetic rate and photosynthate accumulation, which would translate to crop productivity (Koester et al., 2014). In this study, LBS6-treated *Z. mays* showed more absorption of photosynthetic light by the PSII system, and less photosynthetic light was dissipated in the form of heat energy. This was more evident due to higher fraction of PSII open centers in LBS6-treated plants than the control plants. Photosynthetically active energy absorbed by the PSII open centers produces electron gradient through various electron receptors in the thylakoid membranes, which produce ATP and NADPH as an energy source for fixing CO_2_ in the Calvin Cycle (Yamori et al., 2015). LBS6-sprayed *Z. mays* leaves showed a higher rate of flow of electron from antenna complexes to PSII (LEF), the rate of electro-chromic shift (ECS_tau), and steady state rate of proton flux through the chloroplast ATP synthase (gH^+^), which represented a higher rate of ATP synthesis in LBS6-sprayed leaves (Avenson et al., 2004; Ibrahimova et al., 2021). Increase in the photosynthesis-related processes led to better CO_2_ fixation, which was evident by the higher total soluble sugar accumulation in treated leaves. LBS6-treated plants also showed higher total amino acid, total flavonoid, and phenolic contents, suggesting a better metabolic pool to provide enhanced growth, as compared with the control. Shukla et al. (2023) showed that LBS6 regulates the metabolism of carbohydrate to supply energy to the developing cucumber cotyledons (Shukla et al., 2023). Similarly, another type of biostimulant seaweed extract prepared from the wild harvested, brown seaweed *Ascophyllum nodosum* has been known to enhance the growth of corn plants under phosphorous-limited condition by regulating carbohydrate and secondary metabolite metabolism (Shukla and Prithiviraj, 2021). These findings clearly demonstrate that the foliar application of LBS6 improves the growth of plants by differentially regulating physiological and biochemical processes.

### 4.2 The foliar applications of LBS6 modulated soil physiochemical properties, microbial count, and soil enzymes

Increased soil nutrient availability is inversely proportional to soil pH and electrical conductivity (EC). Previously published studies reported that the amendment by the application of biostimulant decreased soil pH and EC, modified soil chemical composition, and improved the assimilation and accessibility of N, P, K, and micronutrients (Yousfi et al., 2021). The present study showed a distinct decrease in soil pH and EC with the growth of those plants sprayed with LBS6. Therefore, the application of LBS6 indirectly contributed to nutrient element enrichment in soil for the betterment of plant growth. Additionally, the results presented in Table 2 also revealed that rhizospheric soil collected from the roots of LBS6-treated plants, showed an increase in the total bacterial and fungal population.

Soil enzymes play essential functions in nutrient cycling and microbial activity, and by studying these enzymes, soil functional health can be correlated with agricultural sustainability (Fei et al., 2020). Most of the metabolic and biochemical reactions occurring in various types of soil are based on the activities of the enzymes produced by microbes (Chen et al., 2020). In line with our assumption, the results presented in this study showed that the rhizospheric soil collected from the roots of plants treated with LBS6 (ST2), which showed modulation of important enzymes, such as FDAse, catalase, and dehydrogenase. Additionally, the soil enzymes related to the nutrient cycle such as urease, aryl sulfatase, and phosphatases were also found to be differentially regulated in ST2. Enhanced activities of FDAse, catalase, and dehydrogenases and increased index of biological fertility were observed in rhizospheric soil grown with LBS6-sprayed plants (Figure 2). Increased FDAse activity in soil represented a higher total microbial activity and their abundance in the soil (Schnurer and Rosswall, 1982), whereas higher dehydrogenase and catalase activity in rhizospheric soil were indicative of higher soil microbial activity, organic matter, and soil fertility (Dotaniya et al., 2018). A similar increase in the activities of soil enzymes was reported in response to the application of *Kappaphycus* sap (Trivedi et al., 2022) and microbe-based biostimulants (Mukherjee et al., 2022).

Microbial populations in various soils play prominent functions in nutrient cycling (Dai et al., 2021). The soil collected from LBS6-sprayed plants showed higher activity of such enzymes involved in nutrient cycling. The urease and phosphatase activities in soil grown with LBS6-sprayed plants (ST2) were found to be higher than in the soil grown with water-sprayed plants (SC2). These results clearly demonstrated that LBS6 enhanced the growth of plants by increasing the nitrogen and phosphorus availability in the soil due to higher urease and phosphatase activities (Koçak, 2020; Margalef et al., 2021). The activity of soil arylsulphatase (ARS), an important enzyme involved in the acquisition of organic sulfur (Chen et al., 2019), was higher in soil from LBS6-sprayed plants. Similar changes in the soil enzymes with the use of other biostimulants were reported in previously published reports (Alam et al., 2013; Wang et al., 2018; Hussain et al., 2021). Thus, changes in these important soil enzymes involved in nutrient cycling, organic carbon sequestration, and fertility of soil grown with the LBS6-treated plants, resulting in better adaptation to the growth conditions.

### 4.3 Foliar spray of LBS6 influenced dynamics of soil microbial diversity

Soil ecological functions are correlated with the diversity and richness in rhizospheric soil (Lehmann et al., 2020). An increase in bacterial diversity, richness, and abundance was observed in rhizospheric soil with LBS6 treatment. Similarly, enhanced richness and abundance due to the application of alkaline extracts from *A. nodosum* and aqueous sap from *K. al*var*ezii* were reported previously (Chen et al., 2022; Trivedi et al., 2022). These findings were also supported by the increased microbial count and enhanced activity of enzymes such as FDAse, dehydrogenase, and catalase in soil. Higher BIF values further demonstrate the role of LBS6 in increasing the microbial abundance in soil.

Further studies reported that the rhizospheric bacterial community undergoes great changes during plant growth, and it largely depends on the root exudates secreted by host plants during the early stages of plant growth (Chen et al., 2022). The soil from the LBS6-sprayed plant showed an abundance of the microbial function involved in chemotaxis (Figure 6D). The microbes expressing chemotaxis proteins such as CheA, CheW, CheX, and CheR (Feng et al., 2021) were found abundantly in soil with LBS6-sprayed plants (ST2), while microbes expressing CheV and MotA (Feng et al., 2021) were abundant in soil with water-sprayed plants (SC2). Important functions in soil related to plant growth such as nitrogen fixation, denitrification, carbon mineralization, and other nutrient cycles were positively correlated with greater bacterial richness and diversity (Fabian et al., 2017; Mhete et al., 2020). Most abundant bacterial phyla present in rhizospheric interactions between plant and soil are members of Proteobacteria (Delgado-Baquerizo et al., 2018; Mukherjee et al., 2022; Trivedi et al., 2022). These bacteria are known to serve general functions in soil and are found to be increased with the growth of soil samples collected from both LBS6 and water-sprayed plants (Malard et al., 2019; Mukherjee et al., 2022). Members of the Alphaproteobacteria belonging to phyla Proteobacteria and Acidobacteria were reported to be positively correlated with nitrogen content of soil (Kristensen et al., 2021; Mukherjee et al., 2022). Plant growth and soil health are significantly influenced by nitrogen metabolism (Tang et al., 2022), spraying of LBS6 increased the richness of Acidobacteria and Alphaproteobacteria, and enhanced activity of urease enzyme is strongly correlated with the plant growth. De-nitrification lowers soil fertility by removing nitrogen, a growth-limiting factor from soil to atmosphere (Rennenberg et al., 2009). Reduction in de-nitrification-related process and enhancement in ureolysis, nitrogen fixation, nitrification, and ammonification indicated that rhizospheric soil of LBS6-sprayed plants showed higher abundance of microbial community involved in nitrogen fixation and maintain soil nitrogen content and fertility. Interestingly, Acidobacteria and Cyanobacteria involved in potassium uptake were abundantly present in soil grown with LBS6-sprayed plants (ST2). The abundance of Latescibacterota, positively associated with total nitrogen, phosphorous, and potassium (Wu et al., 2021), was found to be higher in ST2.

Phosphorus metabolism in soil collected from LBS6-sprayed plants (ST2) was associated with the higher population of Cyanobacteria, Chloroflexi, and Verrucomocrobiota, while in soil collected from the control plants (SC2), the higher P metabolism was due to the abundance of Bacteriodetes. This clearly demonstrates that LBS6 differentially modulated microbial populations involved in P metabolism (Figure 6C). Gammaproteobacteria and Bacteriodetes were mainly associated with the breakdown of organic matter in soil, which was reported to be abundant in soils applied with fertilizers (Franco et al., 2017; Mastný, 2020). Bacteria involved in the decomposition of available organic matter were higher in SC2, while bacteria involved in nutrient metabolism were induced in ST2 by stimulating the richness of beneficial bacterial populations such as Actinobacteria, Acidobacteria, Chloroflexi, and Alphaproteobacteria for better utilization of nutrients.

Stimulation of various metabolic functions including nucleotide, amino acid, co-factors, vitamins, and lipid and energy metabolism was differentially regulated in rhizospheric soils of LBS6-sprayed plants, as compared with the control (Figure 6A; Supplementary Figure S5B). In this study, *K. alvarezii*-derived biostimulant (LBS6) was reported to have considerable impact on root microbiota metabolism (Hussain et al., 2021; Nanda et al., 2022). Stimulation of amino acid and co-factor synthesis pathways, such as IAA production in rhizospheric microbiota through the stimulation of tryptophan biosynthesis, promotes plant growth (Pascale et al., 2020). Our study observed an increase in the abundance of microbes producing chitinolytic and antimicrobial compounds such as streptomycin, tetracycline, and vancomycin, with LBS6 treatment, which indicated the increased biotic stress resistance by the modulation of soil microbial functions (Supplementary Figure S5B) (Pradhan et al., 2022). These results demonstrate that LBS6 improved overall soil health by influencing microbial diversity and functioning.

## 5 Conclusion

This study showed that the foliar application of a particular biostimulant derived from *K. alvarezii* (AgroGain/LBS6) improves morphological and physiological processes in plants, leading to a significant effect on plant growth. The foliar application of LBS6 boosts the capacity of plants to recruit more beneficial microbes by regulating soil enzymatic activities, resulting in a better plant growth. The microbes belonging to the phyla Cyanobacteria, Actinobacteria, Firmicutes, Verrucomicrobiota, Acidobacteria, Bdellovibrionota, Myxococcota, Gemmatimonadota, and Planctomycetota were enhanced in the rhizospheric soil of the plants treated with LBS6. The microbes involved in nitrogen and sulfur metabolism were abundant in soil with LBS6-sprayed plants. Our study highlighted the sustainable impact of the *K. alvarezii* extracts on the soil ecosystem to improve plant growth by maintaining soil health and nutrient balance. This study focused on the changes in the rhizosphere microbial community in *Z. mays* grown in individual pots, which can be different from the plants grown in the field condition. Additionally, more comprehensive and systematic studies are required to study the effect of the biostimulant on the rhizospheric microbial community of plants grown under natural field conditions. We strongly suggest that the judicious use of LBS6 extract as part of a well-managed fertilizer program will reduce the over-dependency on synthetic chemical fertilizers.

## Data availability statement

The datasets presented in this study can be found in online repositories. The names of the repository/repositories and accession number(s) can be found below: BioProject, PRJNA953779.

## Author contributions

NN: Writing – original draft, Software, Methodology, Investigation, Formal analysis, Data curation. PS: Writing – review & editing, Visualization, Validation, Supervision, Resources, Project administration, Methodology, Investigation, Formal analysis, Conceptualization. SN: Writing – review & editing, Visualization, Validation, Supervision, Resources, Project administration, Funding acquisition, Conceptualization. SK: Writing – review & editing, Project administration. SS: Writing – review & editing, Supervision, Resources, Project administration, Funding acquisition, Conceptualization.

## Footnotes

^1^ www.photosynQ.org

^2^ https://www.microbiomeanalyst.ca/

^3^ https://www.ncbi.nlm.nih.gov/biosample/34123503

^4^ https://ccari.icar.gov.in/wasp2.0/index.php

^5^ https://cran.r-project.org/package=pheatmap
