# Supplementary material for: A red seaweed Kappaphycus alvarezii-based biostimulant (AgroGain®) improves the growth of Zea mays and impacts agricultural sustainability by beneficially priming rhizosphere soil microbial community
Source: Front Microbiol. 2024 Apr 2;15:1330237. doi: 10.3389/fmicb.2024.1330237 (PMC11027899; doi:10.3389/fmicb.2024.1330237)

Supplementary Material

A red seaweed *Kappaphycus alvarezii*-based biostimulant (AgroGain®) improves growth of *Zea mays* and impacts agricultural sustainability by beneficially priming soil microbial health.

Nagarajan Nivetha[1], Pushp Sheel Shukla[1*], Sri Sailaja Nori[1], and Shrikumar Suryanarayan[1]

[1]Research and Development Division, Sea6 Energy Private Limited, Centre for Cellular and Molecular Platforms, National Centre for Biological Sciences-Tata Institute of Fundamental Research, Bengaluru, Karnataka, India

***Correspondence:**
Pushp Sheel Shukla, pushp.shukla@sea6energy.com

Supplementary Material

1 Supplementary Figures and Tables

1.1 Supplementary Figures

Supplementary Fig 1. Schematic representation of the experimental design with the sampling time points for plant growth and metagenomic analysis. Base soil sample (BS) was taken before sowing. Maize plants were treated with two doses of LBS6 (+ surfactant) foliar spray and sampled after three and seven days of treatment (ST1 and ST2) and water was used with surfactant for control (SC1 and SC2). BS, SC2 and ST2 samples were used for metagenomics.

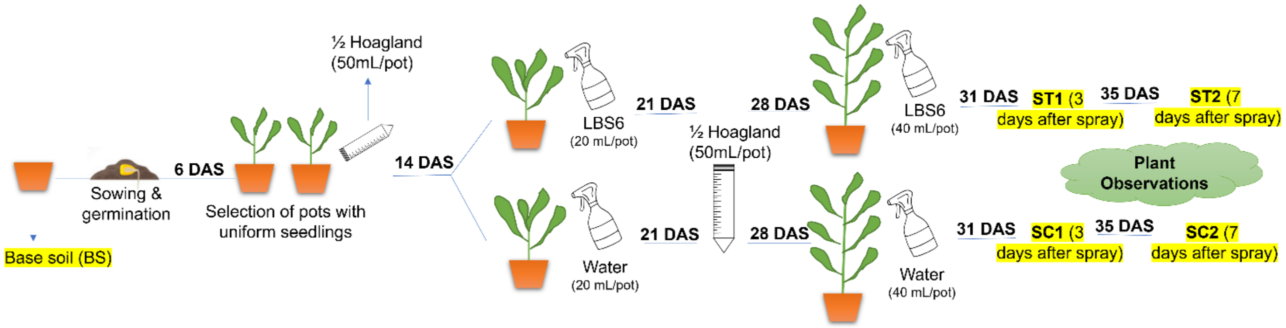

Supplementary Fig 2. Relative bacterial diversity as indicated by (A) Rarefaction curve, (B) Chao1 diversity index, (C) Shannon's diversity index and (D) Simpson's diversity index and (E) Beta diversity. AC1, AC2 and AC3 indicates the replicates of BS, C1, C2 and C3 indicates SC2 and T1, T2 and T3 indicates ST2.

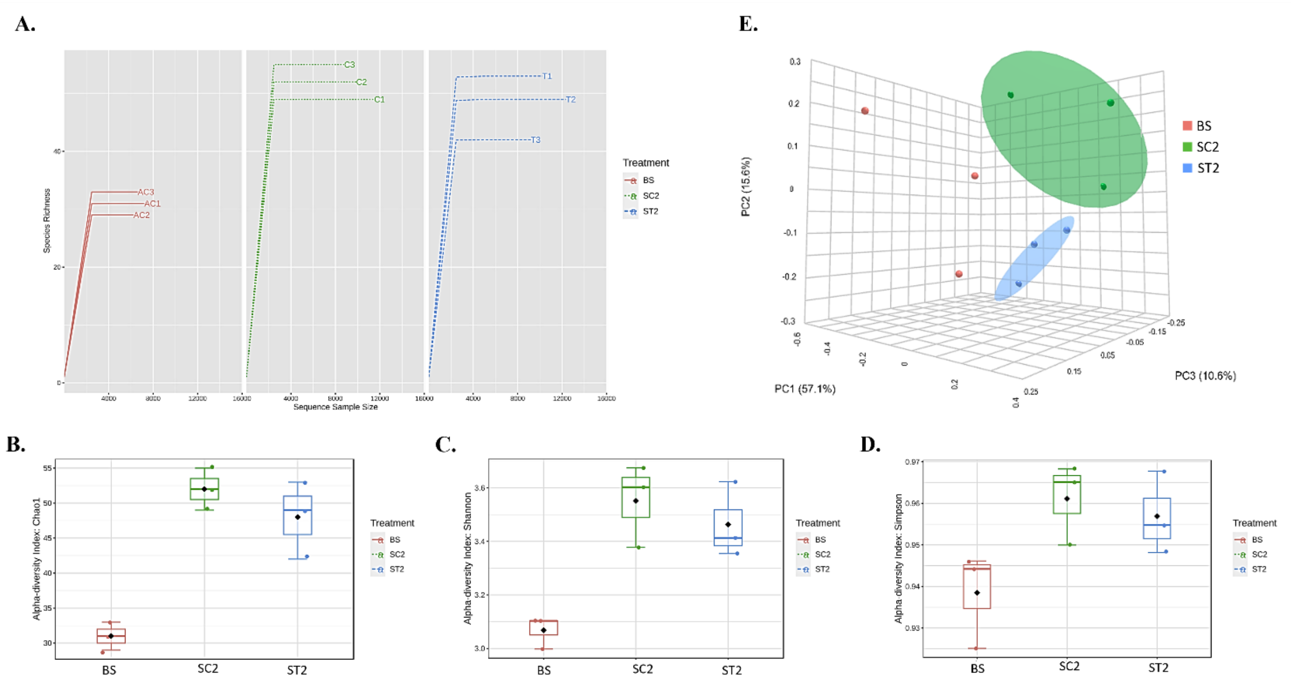

Supplementary Fig 3. Top 16 enriched (A) Phylum, (B) Class and (C) Order categories form all samples (BS, SC2 and ST2).

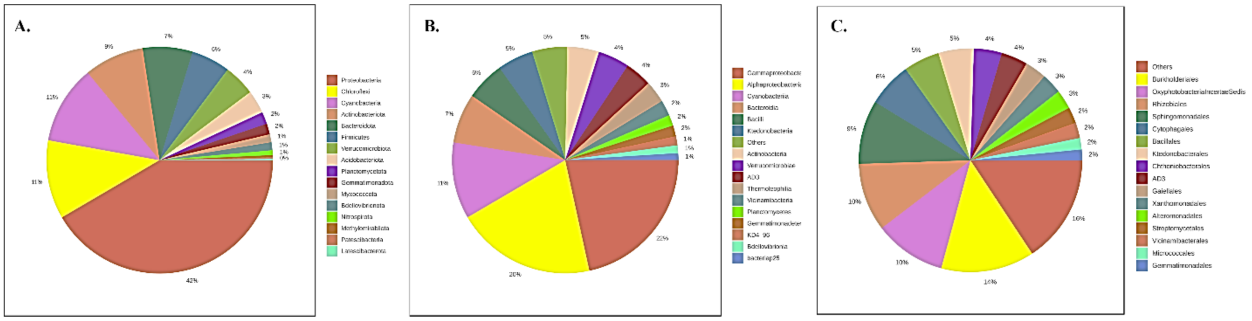

Supplementary Fig 4. Venn diagram to observe the number of unique and shared taxa. (A) Family, (B) Genus and (C) OTUs.

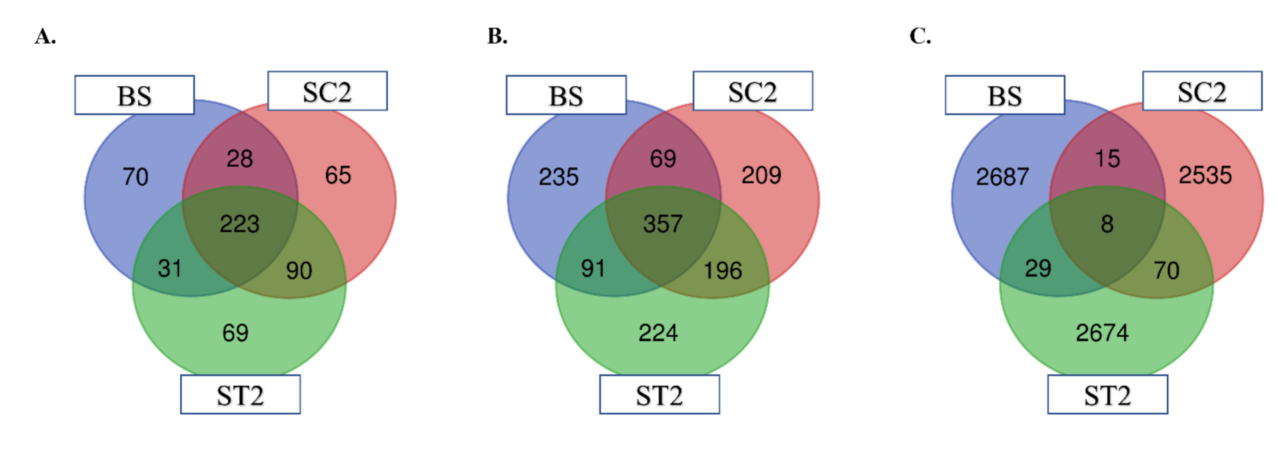

Supplementary Fig 5. LBS6 spray induced differences in the functional profile (A) KEGG Orthologs, (B) KEGG Pathways obtained from TAX4FUN analysis.

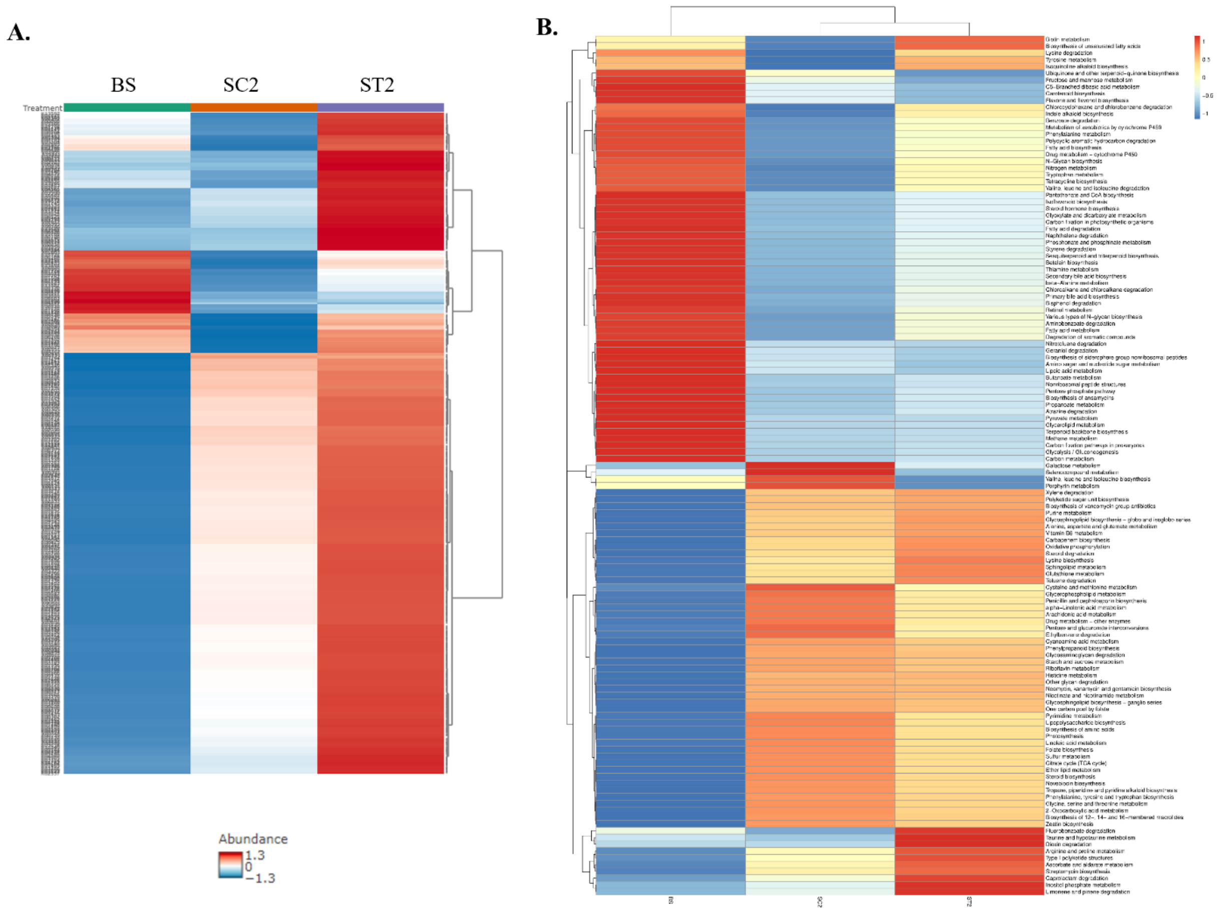

Supplement: Supplementary file 1 [file Data_Sheet_1.pdf]
